# Epidemiology of adult T-cell leukemia/lymphoma (ATL) in people living with HTLV-1: A 30-year study in Peru

**DOI:** 10.1371/journal.pntd.0014010

**Published:** 2026-02-23

**Authors:** Gabriela Garrido-Pinzás, Brian Valenzuela, Elsa González-Lagos, Carlos Seas, Luis Malpica, Carolina Álvarez, Victor Rivera, Fernando Mejía, Martín Montes, Juan Carlos Ramos, Eduardo Gotuzzo

**Affiliations:** 1 Instituto de Medicina Tropical “Alexander von Humboldt”, Universidad Peruana Cayetano Heredia, Lima, Peru; 2 Department of Infectious, Tropical and Dermatological Diseases, Hospital Cayetano Heredia, Lima, Peru; 3 Department of Lymphoma and Myeloma, Division of Cancer Medicine, The University of Texas MD Anderson Cancer Center, Houston, Texas, United States of America; 4 Department of Hemato-Oncology, Hospital Cayetano Heredia, Lima, Peru; 5 Division of Hematology/Oncology, Sylvester Comprehensive Cancer Center, University of Miami Miller School of Medicine, Miami, Florida, United States of America; University of South Florida, UNITED STATES OF AMERICA

## Abstract

**Background:**

Adult T-cell Leukemia/Lymphoma (ATL) is caused by human T-leukemia virus type 1 (HTLV-1). Over 10 million people are infected worldwide, but only up to 5% develop ATL. HTLV-1 infection is endemic in Peru; however, there are currently no reports focusing on the epidemiological characteristics of Peruvian individuals with ATL.

**Methods:**

Data from the HTLV-1 Unit registry covering the period from June 1992 to November 2023 were retrospectively analyzed. Clinical report forms and histopathology records from national referral cancer centers were reviewed. Descriptive statistics were used to characterize patients, and Kaplan-Meier methods assessed survival by ATL subtype.

**Findings:**

A total of 116 confirmed ATL cases were identified. There was a slight female predominance, with 52.6% women (n = 61) and 47.4% men (n = 55). The median age at diagnosis was 54 years (IQR 42–61), with 42.2% of patients diagnosed before age 50. Only 13.8% of patients (n = 16) were diagnosed with HTLV-1 infection before ATL development, and only 8 of those were diagnosed through routine screening. The most common ATL subtype was lymphomatous (65.5%), followed by smoldering/chronic (24.1%), and acute ATL (9.5%). With a median follow-up of 15.9 months, median survival times were 6.5, 12.5, and 89.6 months for acute, lymphomatous, and smoldering/chronic subtypes, respectively. One-year survival rates ranged from 37.5% in acute ATL to 84.6% in smoldering/chronic ATL. Comorbid HTLV-1-associated diseases included infective dermatitis (15.5%), HTLV-1-associated myelopathy/tropical spastic paraparesis (HAM/TSP) (8.6%), and *Strongyloides stercoralis* hyperinfection (6.9%).

**Interpretation:**

This is the first study to describe ATL epidemiology in Peru from an infectious disease perspective. Most patients were unaware of their HTLV-1 status before developing ATL, highlighting missed opportunities for earlier detection. Routine HTLV-1 testing should be considered in the evaluation of T-cell malignancies in endemic countries. In addition, screening high-risk populations could support earlier diagnosis and reduce transmission. Improving access to diagnostic tools, along with stronger collaboration between infectious diseases and oncology services could improve patient outcomes in endemic regions.

## Author summary

Human T-leukemia virus type 1 (HTLV-1) is a virus that infects immune cells and causes a lifelong infection. Discovered more than 40 years ago, it is common in several regions of the world, including parts of South America, the Caribbean, Africa, and Japan. HTLV-1 can be transmitted from person to person through breastfeeding, sexual contact, and blood transfusion. Most people living with HTLV-1 do not experience symptoms or complications. However, a minority develops serious conditions. The most strongly associated diseases include HTLV-1-associated myelopathy/tropical spastic paraparesis (HAM/TSP), a chronic and debilitating neurological disorder that causes progressive weakness in the lower limbs; and adult T-cell leukemia/lymphoma (ATL), an aggressive cancer with a very poor prognosis. There is currently no cure for HTLV-1 infection and no effective treatment for its complications beyond supportive care. Despite its clinical impact, HTLV-1 continues to receive limited public health attention. Here, we describe the epidemiological characteristics of patients diagnosed with ATL over 30 years at the main HTLV-1 referral center in Peru, a country with high HTLV-1 endemicity. We found that most patients did not know they were infected with HTLV-1 until they developed ATL. This highlights the urgent need to raise awareness and expand access to HTLV-1 testing, particularly in endemic settings.

## Introduction

Adult T-cell Leukemia/Lymphoma (ATL) is a rare, aggressive peripheral T-cell malignancy caused by human T-cell leukemia virus type 1 (HTLV-1). The main transmission routes are breastfeeding and sexual intercourse [1,2]. HTLV-1 is endemic in Southwestern Japan, the Caribbean basin, South America, and West Africa. Although more than 10 million people worldwide are infected, less than 5% develop ATL [3].

Clinically, ATL can present indolently (chronic and smoldering subtypes) or aggressively (acute and lymphomatous) [4]. Although the indolent forms have a better prognosis, they may progress to acute ATL, the most aggressive subtype. Clinical features vary by subtype, ranging from asymptomatic leukocytosis or an isolated skin lesion to severe presentations with high fever, altered mental status, and multiorgan failure. ATL cells invade multiple tissues and may lead to organ failure. Because HTLV-1 infects lymphocytes, lymphadenopathy is among the most commonly reported clinical findings, particularly in the aggressive subtypes [1,5,6].

Hypercalcemia and immunosuppression are among the most important complications. Hypercalcemia, which is exclusive to the acute and, less commonly, lymphomatous subtypes, may lead to lytic bone lesions and renal impairment. Immunosuppression predisposes patients to opportunistic, life-threatening infections [5].

The age of ATL presentation and clinical subtype varies widely between regions, possibly influenced by host factors and disparities in early healthcare access [7–11]. As of 2025, ATL remains incurable primarily with conventional chemotherapy, with no global treatment consensus [8].

Peru is an epicenter for HTLV-1 infection, with a reported seroprevalence in adults of up to 3.8% [12]. Since most patients with HTLV-1 infection are asymptomatic and screening in Peru is limited to blood donors, diagnosis typically follows the development of an HTLV-1-associated disease. ATL may clinically and histologically mimic cutaneous and T-cell lymphomas such as Mycosis Fungoides (MF) and Sézary syndrome; thus, even when these malignancies develop, HTLV-1 infection may remain undiagnosed [6,13,14]. To date, little information has been published regarding ATL in the Peruvian population, and oncology teams have performed most studies [8,15–17]. We aim to describe the epidemiological characteristics of HTLV-1 infection and ATL based on cases encountered over the past 30 years in a major Peruvian referral center.

## Methods

### Ethics statement

The Institutional Review Boards of Universidad Peruana Cayetano Heredia (UPCH) and Hospital Cayetano Heredia (HCH) approved the study protocol. The need for informed consent was waived because the information was obtained retrospectively, and patients’ data were anonymized.

We conducted our study in Lima, Peru, at two locations: (1) the HTLV-1 Unit of the Instituto de Medicina Tropical “Alexander von Humboldt” (IMTAvH), Universidad Peruana Cayetano Heredia (UPCH), the country’s leading referral center for patients with HTLV-1 infection; and (2) Hospital Cayetano Heredia (HCH), a major referral hospital adjacent to IMTAvH. Patients referred to the HTLV-1 Unit include those with a positive HTLV-1 screening test identified during routine blood bank evaluations, suspected HTLV-1-associated diseases, or seropositive family members. Although the HTLV-1 Unit functions as a national referral center for HTLV-1 infection, ATL is diagnosed and managed in multiple institutions across the country, and only a subset of patients are ultimately referred to our center. All patients were initially assessed by trained healthcare professionals who provided pre-test counseling. Those diagnosed with HTLV-1 infection received ongoing medical consultations with clinicians who specialize in the management of HTLV-1.

We reviewed all case report forms (CRFs) documenting ATL, irrespective of histologic confirmation or timing of diagnosis, among HTLV-1 seropositive patients who attended the unit between June 1992 and November 2023.

### Definition of main variables

Confirmed ATL diagnosis was defined by the presence of anatomopathological and/or flow cytometry findings consistent with T-cell lymphoma or leukemia, in addition to positive confirmatory testing for HTLV-1 infection using Western Blot (WB), quantitative and/or qualitative polymerase chain reaction (PCR), or Immunoblot Line Assay (INNO-Lia).

The date of ATL diagnosis was established by the date of confirmation of the evidence. The date of HTLV-1 diagnosis corresponded to the first reactive HTLV-1 test result. A simultaneous occurrence of ATL and HTLV-1 diagnosis was defined when both diagnoses were within 90 days of each other.

For ATL subtypes classification (acute, lymphomatous, and smoldering/chronic subtypes), we followed Shimoyama criteria, conditioned on data availability (15.3%, n = 34): otherwise, we transcribed the subtypes as recorded in the CRFs. Because of histopathological overlap and HTLV-1/ATL potential underdiagnosis, we also classified as “smoldering/chronic” ATL the cases reported exclusively with cutaneous lesions, indolent course, and with “Cutaneous T-cell lymphoma” or “Mycosis fungoides”.

Mainly based on the HTLV-1 status of family contacts of our study population, we defined the presumed transmission route. The breastfeeding route was determined when cases (1) did not deny being breastfed, and (2) their tested mother was seropositive; in the absence of maternal results, we considered the seropositivity of the father or siblings. The sexual route was determined for cases with (1) an HTLV-1 positive sexual partner, (2) an HTLV-1 negative mother and/or no history of breastfeeding, and (3) no history of blood transfusions. Blood transfusion route was determined for cases with (1) an HTLV-1 negative mother or no history of breastfeeding, (2) HTLV-1 negative sexual partner, and (3) a history of blood transfusion before 1998, when mandatory HTLV-1 blood donor screening was locally implemented. We classified the transmission route as indeterminate when none of the criteria were met.

Ethnicity was defined as “Quechuan” when the reported parental language was Quechua; in other cases, we used the self-reported ethnicity. The duration of breastfeeding was also self-reported. Comorbidities were determined based on laboratory results and medical records attached to the CRFs or self-reported. Survival time was calculated from the date of ATL diagnosis to the date of death or last follow-up at the study center.

**Fig 1:**
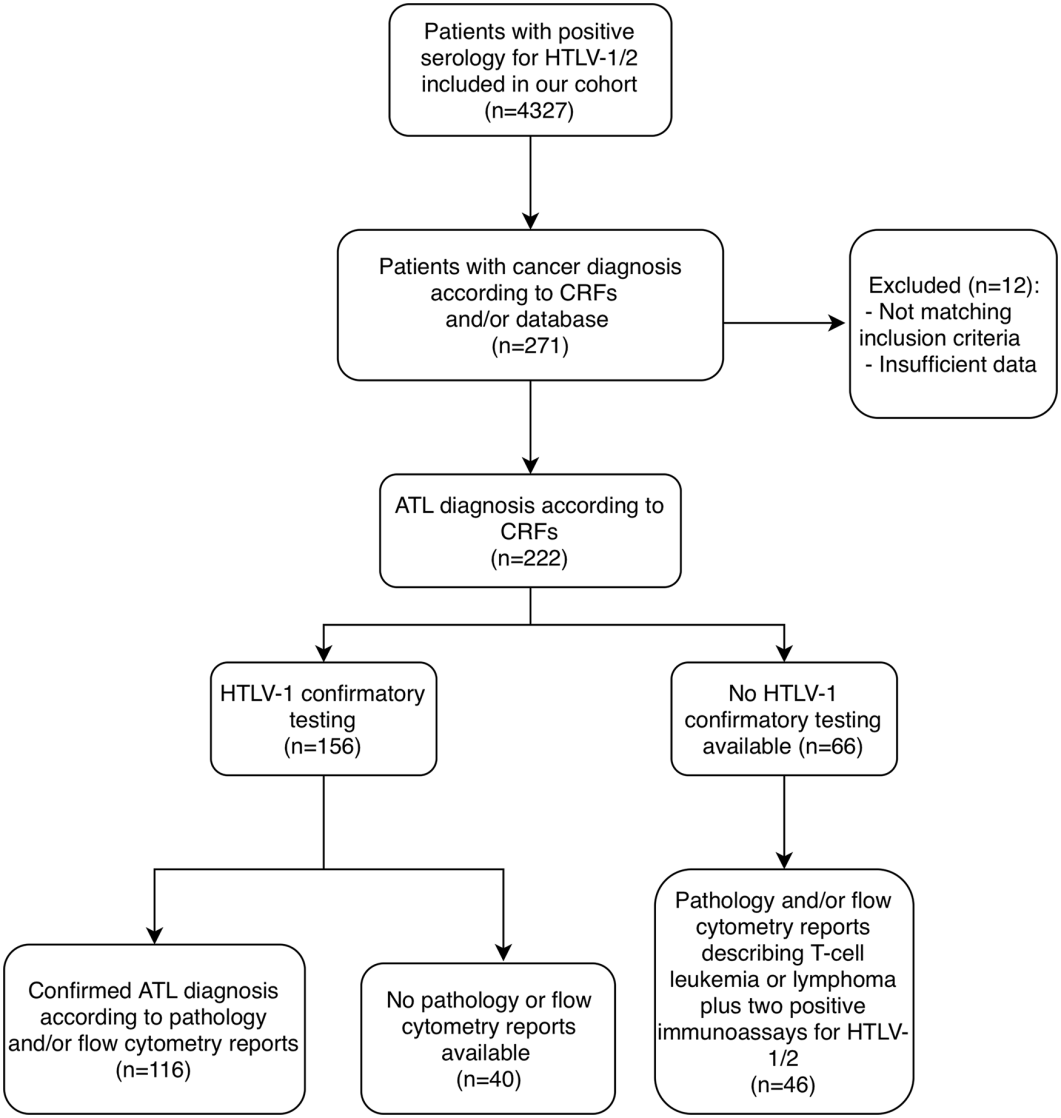
Flowchart for patient assessment.

To acknowledge potential diagnostic gaps or incomplete diagnoses, we identified a secondary group of patients with clinical diagnoses suggestive of ATL but lacking sufficient laboratory confirmation (no confirmatory HTLV-1 testing and/or definitive histopathologic or flow cytometry evidence). These cases were documented separately to highlight diagnostic gaps and potential underestimation of ATL and other cancers’ prevalence. Fig 1 provides a detailed flowchart summarizing patient identification and inclusion/exclusion criteria for confirmed ATL cases and clinically diagnosed but unconfirmed ATL cases.

### Data management and analysis

Data were primarily collected from handwritten CRFs at the HTLV-1 Unit, histopathology records from referring cancer centers provided by patients, and additional clinical and laboratory data from routine Excel and Access databases. Within our main referral hospital (HCH), we accessed outpatient oncology clinic records and Pathology Department computer archives. We extracted information on age at HTLV-1 and ATL diagnosis, sex, ethnicity, family history of HTLV-1 infection, breastfeeding duration, history of blood transfusion, ATL subtypes (acute, chronic, lymphomatous, smoldering), and other HTLV-1-associated diseases (HAM/TSP, IDH, SHS). Data were entered into a secure REDCap database, where unique research IDs were assigned and personal identifiers were removed post-extraction to ensure confidentiality. Data were checked for duplicates and inconsistencies, with database access restricted to the research team.

Our primary analysis focused exclusively on confirmed ATL cases. Descriptive analyses were also performed for unconfirmed ATL cases. We analyzed data using the Stata software, version 14 (StataCorp., College Station, TX, USA), and R, version 4.5.0 (R Foundation for Statistical Computing, Vienna, Austria). Descriptive analyses included measures of central tendency and dispersion for continuous variables, and percentages and absolute values for categorical variables. Additionally, we conducted a survival analysis to compare the overall survival rates among different ATL subtypes. We used the Kaplan-Meier method to estimate survival curves and the log-rank test for differences between subgroups. Statistical significance was defined as a p-value of less than 0.05. Median survival times and 95% confidence intervals (95% CI) were reported. Confidence intervals affected by censoring were estimated via bootstrap resampling.

## Results

The flowchart for patient assessment and inclusion is shown in Fig 1. Of the 4327 seropositive patients registered at the HTLV-1 Unit, 116 cases with confirmed ATL diagnosis were identified and included in our study (Table 1). An additional 106 patients with ATL diagnoses reported by their attending physicians on CRFs were excluded due to lack of available documentation to support the diagnosis. There was a slight predominance of female patients (n = 61; 52.6%) over males (n = 55; 47.4%). Age at diagnosis was available for 115 patients (99.1%), as one patient lacked a recorded date of birth. The median age at ATL diagnosis was 54 years (IQR 42–61), and 42.2% of patients (n = 49) were 50 years or younger at the time of ATL diagnosis. Most patients were either Mestizo (n = 56; 48.3%) or Quechuan (n = 51; 44.0%).

**Table 1:** Demographic, clinical and epidemiological characteristics of patients with Adult T-cell Leukemia/Lymphoma (N = 116).

	n (%)
Age at ATL diagnosis (years)*	n = 115 54 [42-61]
Timing ATL-HTLV-1 diagnoses	n = 116
Diagnosed with HTLV-1 infection prior to ATL diagnosis (>90 days)	16 (13.8)
Diagnosed with HTLV-1 infection within 90 days of ATL diagnosis	83 (71.5)
Diagnosed with HTLV-1 infection after ATL diagnosis (>90 days)	17 (14.7)
Sex at birth	
Female	61 (52.6)
Male	55 (47.4)
Ethnicity	
Mestizo	56 (48.3)
Quechuan	51 (44.0)
Asian	4 (3.5)
Afro-Peruvian	2 (1.7)
Unspecified	3 (2.6)
Comorbidities	
Infective dermatitis	18 (15.5)
Tuberculosis infection	8 (6.9)
*Strongyloides stercoralis* hyperinfection	8 (6.9)
Crusted scabies	6 (5.2)
HAM/TSP	10 (8.6)
Uveitis	1 (0.9)
Presumed route of transmission	
Breastfeeding	29 (25.0)
Sexual intercourse	5 (4.3)
Blood transfusion	1 (0.9)
Indeterminate	81 (69.8)
ATL subtype	
Lymphomatous	76 (65.5)
Smoldering/chronic	28 (24.1)
Acute	11 (9.5)
Unknown/ Unspecified	1 (0.9)

ATL, Adult T-Cell Leukemia/Lymphoma; HTLV-1, Human T-Lymphotropic virus type 1; HAM/TSP: HTLV-1-associated myelopathy/Tropical spastic paraparesis.

*Median [IQR]

The most likely transmission routes of HTLV-1 were breastfeeding (n = 29; 25.0%); sexual intercourse (4.3%; n = 5); and blood transfusion (0.9%; n = 1). The transmission route was not determined in 69.8% of patients (n = 81) due to insufficient data. The single patient with a confirmed ATL diagnosis whose transmission route was presumed to be blood transfusion had a mother and wife who tested negative for HTLV-1, denied having any other sexual partners, and had received a blood transfusion at one year of age, before mandatory HTLV-1 blood donor screening in Peru. ATL developed in this patient 28 years after the said transfusion.

### Clinicopathological features and outcomes of ATL patients

The most common ATL subtype reported was lymphomatous ATL (n = 76; 65.5%), followed by smoldering/chronic (n = 28; 24.1%) and acute ATL (n = 11; 9.5%). The ATL subtype was not determined in 0.9% of cases (n = 1).

Forty-six patients underwent bone marrow biopsy, and 27 (58.7%) were reported to have bone marrow involvement. Notably, bone marrow involvement was not always confirmed by flow cytometry or immunohistochemistry.

Skin involvement was reported in 66.7% of patients (n = 50/75) of whom 21 (42.1%, n = 21/50) had anatomopathological features resembling MF. A history of IDH was reported by 18 patients (15.5%), of whom 12 presented skin lesions at the time of ATL diagnosis. Additionally, eight patients (6.9%) had a history of *Strongyloides stercoralis hyperinfection syndrome* (SHS), 10 (8.6%) HTLV-1-associated myelopathy/tropical spastic paraparesis (HAM/TSP), 8 (6.9%) tuberculosis infection, 6 (5.2%) crusted scabies, and 1 (0.9%) HTLV-1-associated uveitis.

Eighty-three patients (71.5%, n = 83/116) were tested for HTLV-1 infection following the initial anatomopathological and/or flow cytometry report or up to 90 days before their diagnosis. Only 16 patients (13.8%, n = 16/116) were diagnosed with HTLV-1 infection prior to the development of ATL. Four patients were initially diagnosed due to HAM/TSP, and two due to SHS. Only 8 patients were diagnosed during routine screening: 4 due to positive family history, and 4 due to positive blood bank screening.

**Fig 2:**
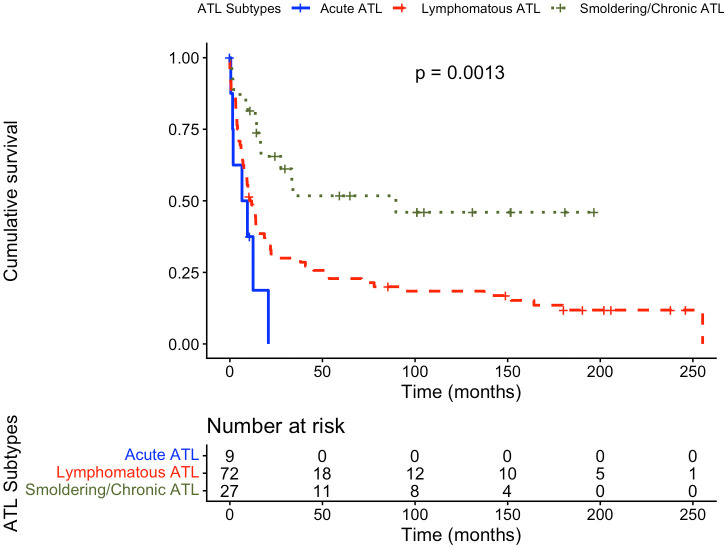
Kaplan-Meier Survival curves for patients with Adult T-Cell Leukemia/Lymphoma diagnosis. ATL, Adult T-Cell Leukemia/Lymphoma.

We conducted a survival analysis to compare the overall survival rates of patients with ATL according to their subtype. With a median follow-up time of 151.9 months (95% CI: 101.9–202.1), the median survival times were 6.5 months (95% CI: 1.5–12.5) for acute ATL, 12.5 months (95% CI: 4.8–53.6) for lymphomatous ATL, and 89.6 months (95% CI: 16.8–not reached) for smoldering/chronic ATL. The overall median survival time for all ATL patients, regardless of subtype, was 14.4 months (95% CI: 6.0–94.3). Median survival times differed significantly among subtypes (log-rank χ² = 13.2, p = 0.001) (Fig 2). One-year survival rates were 37.5% (95% CI: 8.7–67.4) for acute ATL, 51.4% (95% CI: 38.9–62.5) for lymphomatous ATL, and 84.6% (95% CI: 64.0–93.9) for smoldering/chronic ATL.

### Patients with unverified ATL diagnoses

A total of 106 patients were reported to have ATL but were excluded from our final analysis because their diagnosis could not be verified through available documentation: HTLV-1 confirmatory testing records were unavailable in 66 cases, and pathology and/or flow cytometry reports in 40 cases.

Fifty-seven patients (53.8%) were female, while 49 patients (46.2%) were male. The most frequent ATL subtype according to CRF descriptions was lymphomatous (64.2%, n = 68), followed by smoldering/chronic (18.9%, n = 20) and acute ATL (16.0%, n = 17). The median age at ATL diagnosis was 58 years (IQR 48–70). Most patients were identified as Mestizo (48.1%) or Quechuan (49.1%). The probable HTLV-1 transmission routes were breastfeeding in 25.5%, sexual contact in 4.7%, and indeterminate in 69.8% of cases. In terms of comorbidities, 9.4% had a history of infective dermatitis, 7.6% had tuberculosis, and 7.6% had crusted scabies. Only one patient (0.9%) was also diagnosed with HAM/TSP.

## Discussion

In this 30-year retrospective study, we identified 116 confirmed cases of ATL, with a median age at diagnosis of 54 years, and 42.2% of patients diagnosed before age 50. The lymphomatous subtype was the most common, followed by indolent forms, which appeared more frequently than previously reported in Latin American cohorts. Nearly two thirds of evaluable patients presented with skin involvement at onset. Despite these findings, overall survival was poor, with a median survival of 14.4 months across all subtypes and as low as 6.5 months for patients with acute ATL.

Only 13.8% of patients (n = 16/116) were diagnosed with HTLV-1 infection prior to ATL development. In Peru, HTLV-1 testing remains limited to blood donors and is rarely integrated into routine care. As a result, most are tested only after symptoms of HTLV-1-associated diseases appear—and even then, diagnosis may be missed if clinicians do not recognize its clinical features. This limits opportunities for prevention, such as promoting safe sex practices, discouraging breastfeeding in infected mothers, and vaccinating high-risk populations once vaccines become available. We believe broader HTLV-1 testing among pregnant women and first-degree relatives of infected individuals could support earlier diagnosis and timely intervention.

Although the lifetime risk of ATL is reportedly higher in men, we observed a slight female predominance (52.6%) in our cohort [18]. Similar trends have been reported in Latin America, Brazil, the USA, and Spain [6,8–10,19]. The median age of ATL diagnosis was 54 years (IQR 42–61)**,** which aligns with previous reports [7,9,10]. A recent retrospective study across multiple Latin American countries reported a median diagnosis age of 57 years [8]. However, lower median ages at diagnosis have been reported in Brazil (49 years), Spain (37 years), South Africa (47 years), and French Guiana (48 years) [6,13,19–21]. In Japan, the most endemic country, the onset of disease is later, at around 69 years of age [11,22,23]. The higher age at diagnosis in Japan has been attributed to the overall aging of the population, which in turn leads to more ATL cases being diagnosed in older adults [11].

The most common ATL subtype described was lymphomatous ATL (65.5%). Although to a lesser extent, the lymphomatous subtype has also been reported as predominant in Latin America (48.5%), Spain (54.3%), and Florida (49.2%), where a large percentage of the population is Hispanic, as well as in French Guiana (58%) [7,8,19,21]. In contrast, Brazil, Japan, South Africa, and New York have all reported the acute subtype as predominant [6,9,10,20,22]. After lymphomatous ATL, smoldering/chronic ATL was the most prevalent subtype, with 24.1% of patients exhibiting indolent forms of ATL. A high percentage of patients with an indolent form of ATL has also been reported in Brazil, where 27.0% of patients presented with the smoldering subtype [13,24]. The authors attribute this to having dermatologists as part of their clinical team, which is the case in our institution.

Cutaneous involvement was a prominent clinical feature in our cohort (66.7%, n = 50/75)**.** Higher rates have been reported in Brazil (67%), while North American and Latin American cohorts report lower rates, ranging from 21.7% to 30% [6,8,9]. Twenty-one (42.1%) patients with skin lesions had an MF-like pattern on anatomopathology. Oliveira et al. reported that, among their patients, 28.6% of them had an MF-pattern. This histopathological pattern has been associated with a higher survival when compared to peripheral T-cell lymphoma unspecified (PTCL-U), and it is commonly associated with an indolent form of ATL [6,13,14]. Based on these findings, we recommend that in HTLV-1 endemic areas, dermatopathologists should be aware of the sometimes indistinguishable histopathological features of ATL compared to other cutaneous and peripheral T-cell lymphomas [13].

In a multicenter series, Malpica et al. described 159 ATL cases from three major hospitals (Instituto Nacional de Enfermedades Neoplásicas, Hospital Nacional Edgardo Rebagliati Martins, and Hospital Nacional Guillermo Almenara), describing lymphomatous ATL as the most common subtype (62.9%), followed by acute ATL (24.5%) [8]. The lower proportion of acute ATL in our cohort (9.5%) may reflect referral patterns, as many patients with acute ATL are severely ill and remain in the centers where they are initially diagnosed. Additionally, the close proximity of the Dermatology Department at Hospital Cayetano Heredia to our unit results in many referrals originating from this service, potentially contributing to the higher frequency of cutaneous involvement in our series. Overall, our cohort may not reflect the national spectrum of ATL, and broader multicenter studies are needed.

One hundred and six patients with reported ATL were not included in the primary analysis because their diagnoses could not be verified through available documentation. Similarly to the group included in the final analysis, this group showed a slight female predominance (53.8%), and the predominant subtype was lymphomatous ATL (64.2%) followed by smoldering/chronic ATL (18.9%) and acute ATL (16.0%). The median age at diagnosis was slightly higher (58 years). These comparable findings underline that, in endemic and resource-limited settings, the combination of a T-cell malignancy and a reactive HTLV-1 screening immunoassay strongly support the diagnosis of HTLV-1 infection. In our population, ELISAs have shown excellent positive predictive values (98–100%), with false-negative rather than false-positive results being the greater concern [25]. Thus, in endemic regions, we recommend that clinicians consider two reactive HTLV-1 immunoassays as sufficient to confirm HTLV-1 infection when confirmatory testing is unavailable, and use this information to help guide early clinical decision-making.

### Other HTLV-1-associated diseases

#### HTLV-1-associated myelopathy/tropical spastic paraparesis (HAM/TSP).

HTLV-1-associated myelopathy/tropical spastic paraparesis (HAM/TSP) is a chronic, progressive neuroinflammatory disorder that primarily affects the spinal cord, leading to symmetric paraparesis in the lower limbs with signs of pyramidal tract involvement [26]. In addition to the classic pyramidal signs, lower limb paresthesias, urinary complaints, constipation, and lumbar pain are among the most frequently reported manifestations [27].

It predominantly affects women, and most patients experience a slowly progressive course, with a median of 19 years before bilateral walking assistance becomes necessary. As with ATL, HAM/TSP has no definitive treatment besides symptomatic management [27,28].

In our study, 8.6% of patients had HAM/TSP. This association has been described as uncommon [29]. Other studies report varying prevalence rates, with 7.8% in Spain and 3% in Florida, while Brazil reports a higher prevalence of 13.3% to 14.0% [6,7,19]. In a six-year prospective study conducted in Peru, none of the HAM/TSP patients either developed or had a history of ATL [30].

#### Infective dermatitis associated with HTLV-1 (IDH).

Infective dermatitis associated with HTLV-1 (IDH) is a severe form of chronic eczema characterized by recurrent *Staphylococcus aureus* or beta-hemolytic *Streptococcus* skin infections. It typically manifests during childhood but may occasionally occur in adults. Clinically, it presents with erythematous, scaly, and pruritic lesions that most commonly involve the scalp and retroauricular areas.

In our study, 18 patients (15.5%) had a history of IDH, and most (n = 12/18) presented with skin lesions at ATL onset. Progression from IDH to ATL has been described [6]. Bittencourt et al. reported that 37.5% of patients with ATL and skin lesions had a history of IDH [14,31]. In our study, among the 50 patients with skin involvement**,** 12 (24.0%) had a history of IDH. Because most IDH cases present during childhood, and because it is frequently misdiagnosed, its frequency in our cohort may be underestimated.

The viral genotype may influence the clinical presentation of HTLV-1 infection. Most cases of ATL and HAM/TSP have been described in regions where the cosmopolitan a-genotype (HTLV-1a) predominates [32]. In contrast, bronchiectasis appears to be a much more frequent manifestation among patients infected with the Australo-Melanesian c-genotype (HTLV-1c) [33,34]. The HTLV-1c genotype has been proposed to be less strongly associated with malignant transformation, possibly due to the absence of the p30 region, which may contribute to T-cell transformation in patients with the HTLV-1a strain.

Because HTLV-1a is the predominant genotype in Peru, ATL and HAM/TSP are the most frequently observed HTLV-1-associated diseases [35]. Bronchiectasis has been reported as an HTLV-1 complication among Peruvian patients without prior tuberculosis infection, although data on this complication is limited [36].

Our study’s retrospective design posed limitations. Shimoyama subtype classification could not be consistently applied due to missing laboratory data in older case records. Information on comorbidities and ethnicity was based on self-reported data from historical records, which may have introduced some misclassification. In addition, as a single-center study, our sample may be subject to selection bias and may not fully represent ATL in Peru. Despite these limitations, our study has several strengths. The use of systematically collected data over three decades in a national HTLV-1 referral center allowed us to describe not only the clinical and epidemiological patterns of ATL, but also HTLV-1–associated comorbidities, likely transmission routes, and diagnostic delays. Our findings can help inform future screening and prevention strategies in areas endemic for HTLV-1.

In conclusion, this study provides a comprehensive overview of ATL among HTLV-1-infected patients in Peru, based on a 30-year clinical cohort. Our findings highlight the need for enhanced HTLV-1 screening and early detection strategies. In endemic settings, integrating HTLV-1 testing into oncology and dermatologic evaluations could improve case identification. Screening high-risk populations may also contribute to timely diagnosis and prevention. Further controlled prospective studies with larger populations are needed.
